# Supplementary material for: Porous Carbon Spheres Derived from Hemicelluloses for Supercapacitor Application
Source: Int J Mol Sci. 2022 Jun 26;23(13):7101. doi: 10.3390/ijms23137101 (PMC9267052; doi:10.3390/ijms23137101)
Supplement: Supplementary file 1 [file ijms-23-07101-s001.zip › ijms-1777611-supplementary.pdf]

Supporting Information

Porous carbon spheres derived from hemicelluloses for supercapacitor application

Yuanyuan Wang[1], Chengshuai Lu[1], Xuefei Cao[1,2,*], Qiang Wang[1], Guihua Yang[1], Jiachuan Chen [1,*]

[1] *State Key Laboratory of Biobased Material and Green Papermaking, Qilu University of Technology, Shandong Academy of Sciences, Jinan, 250353, China*

[2] *Beijing Key Laboratory of Lignocellulosic Chemistry, Beijing Forestry University, Beijing, 100083, China*

*Authors to whom correspondence should be addressed.

E-mail: caoxuefei@bjfu.edu.cn (Xuefei Cao)

Tel.: +86-010-62336903; Fax: +86-010-62336903.

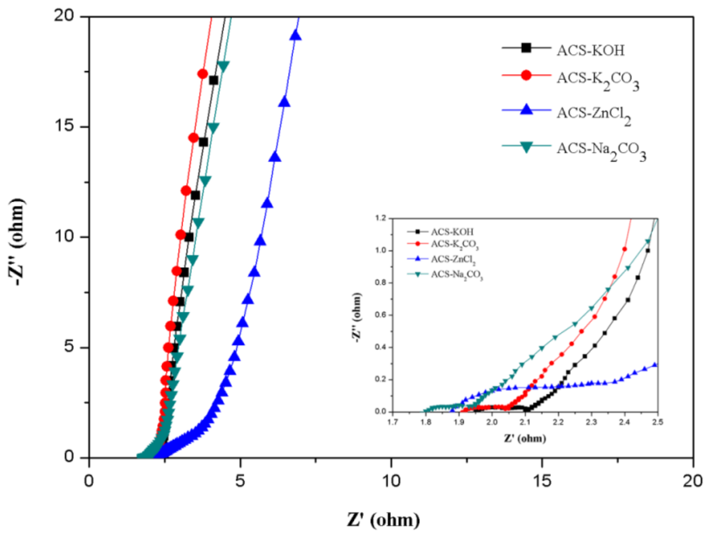

Figure S1 Nyquist plots of ACS-Na_2CO_3, ACS-KOH, ACS-K_2CO_3, and ACS-$ZnCl_2$ in 6 M KOH in three-electrode system
